# Evaluating the impact of equity focused health impact assessment on health service planning: three case studies

**DOI:** 10.1186/1472-6963-14-371

**Published:** 2014-09-05

**Authors:** Ben Harris-Roxas, Fiona Haigh, Joanne Travaglia, Lynn Kemp

**Affiliations:** Centre for Primary Health Care and Equity, University of New South Wales, Sydney, NSW 2052 Australia; School of Public Health and Community Medicine, University of New South Wales, Sydney, NSW 2052 Australia

**Keywords:** Health impact assessment, Health equity, Health service planning, Impact evaluation, Evaluation framework, Conceptual framework

## Abstract

**Background:**

Health impact assessment has been identified internationally as a mechanism to ensure potential health impacts and health equity impacts of proposals are considered before implementation. This paper looks at the impact of three equity focused health impact assessments (EFHIAs) of health service plans on subsequent decision-making and implementation, and then utilises these findings to test and refine an existing conceptual framework for evaluating the impact and effectiveness of health impact assessments for use in relation to EFHIAs.

**Methods:**

Case study analysis of three EFHIAs conducted on health sector plans in New South Wales, Australia. Data was drawn from 14 semi-structured interviews and the analysis of seven related documents (draft plans and EFHIA reports).

**Results:**

The case studies showed that the EFHIAs all had some impact on the decision-making about the plans and their implementation, most clearly in relation to participants’ understandings of equity and in the development of options for modifying service plans to ensure this was addressed. The timing of the EFHIA and individual responses to the EFHIA process and its recommendations were identified as critical factors influencing the impact of the EFHIAs. Several modifications to the conceptual framework are identified, principally adding factors to recognise the role individuals play in influencing the impact and effectiveness of EFHIAs.

**Conclusion:**

EFHIA has the potential to improve the consideration of health equity in health service planning processes, though a number of contextual and individual factors affect this. Current approaches can be strengthened by taking into account personal and organisational responses to the EFHIA process.

**Electronic supplementary material:**

The online version of this article (doi:10.1186/1472-6963-14-371) contains supplementary material, which is available to authorized users.

## Background

The use of health impact assessment (HIA) has expanded rapidly over the past twenty years [1–5]. HIA is a stepwise process for assessing the potential health impacts of a range of different types of proposals, including plans, projects, policies or programs. It seeks to assist decision-making and implementation of proposals by developing evidence-informed recommendations to maximise positive health impacts and to minimise negative ones [6–12]. HIAs’ recommendations can take several forms and may include measures designed to:

- mitigate potentially negative health impacts [8];
- enhance potentially positive health impacts [13];
- improve the distribution of potential health impacts within and between population sub-groups [10, 14–16];
- promote alternative approaches that are designed to achieve similar policy or program objectives [1, 13, 17]; or
- recommend that the proposal should not proceed [18].

There is a broad consensus that HIA is most useful and has the greatest potential to influence decision-making and implementation when it is conducted as an ex ante assessment prior to the implementation of a proposal [10, 13, 19–21].

### Equity focused health impact assessment

Equity focused health impact assessment (EFHIA) is a specific form of HIA and has been promoted by public health organisations regionally, nationally and internationally. It is one of a number of strategies to ensure health equity is considered in the development of policies, programs and plans [14, 15, 22–32].

EFHIA follows health impact assessment processes to firstly determine the potential differential and distributional impacts of a proposal on the population as well as specific groups within that population and secondly, to assesses whether the differential impacts are inequitable. The equity dimension of EFHIA is about assessing whether identified differential health impacts are inequitable, i.e. the result of factors that are avoidable and unfair and potentially preventable or avoidable [15]. The EFHIA Framework was published in Australia in 2004 and has subsequently been used in Australia and internationally [33–35] and has informed the development of related approaches such as health equity impact assessment [28, 36, 37].

Though all HIAs should consider health equity, vulnerabilities and the distribution of potential impacts [38] in practice this aspiration has been difficult to realise [16, 22, 23, 36], often because it adds a layer of complexity to already time- and resource-constrained assessment processes [1]. EFHIA has been developed as a distinct form of HIA to provide a structured process for assessing health equity impacts.

EFHIA is only one of a number of interventions that aim to ensure health equity issues are addressed in planning and implementation. A recent review by the University of Victoria in Canada identified a total of 36 health equity-focused tools that are designed to inform needs assessment, planning, impact assessment, implementation and evaluation [39, 40]. HIA and EFHIA are amongst the best described and most researched of the health equity tools identified. The findings of this study may have relevance to these other equity-focused tools.

### HIA of health sector proposals

HIA has historically been principally regarded as a procedure and tool to promote inter-sectoral action for health [25, 41–45], for example calls for its use in *The Ottawa Charter* and the WHO Commission on the Social Determinants of Health’s final report [24, 46]. Most HIAs have focused on sectors such as land use planning, transport and social policy proposals rather than health sector policies, plans and programs [5]. Despite this trend, HIAs are also conducted on health sector proposals [47–52].

There has been a recognition amongst researchers and policy-makers that even though HIA may be most used in inter-sectoral settings, there is still value in assessing the population-level impacts of health sector initiatives [1, 53]. This is because while health sector plans explicitly seek to address health needs and health outcomes, they may not have fully considered impacts on health equity for a number of reasons. These may include the lack of opportunities to examine differential impacts within and between population sub-groups during planning and policy development, or time to consider how aspects of the design and implementation of health sector proposals could exacerbate health inequalities and increase the social gradient in health [32] by benefitting healthy people more than those with poor health [15, 50].

A good example of the recognition for this need to look at health sector initiatives comes from the setting for this study. The New South Wales *Health and Equity Statement* from 2004 called for the development of “a process for undertaking Rapid Health Impact Appraisals within NSW Health to identify the health impact of existing and new policies” [54]. This was distinct from more comprehensive approaches to HIA that the *Statement* recommended be used intersectorally. EFHIA, in particular ones that are conducted rapidly, have been recommended as a mechanism to address this need [16, 22, 24, 25, 28, 42, 55].

### The need to demonstrate effectiveness

There have been calls for research to focus on the effectiveness of HIA and EFHIA if its use is to become more widespread and to justify investment in this process [1, 56–61]. Health systems and governments are resource-constrained, and interventions are increasingly expected to demonstrate their utility [24, 62]. Whilst there are a growing number of case studies demonstrating HIA’s effectiveness in various contexts [63–75] it is still unclear whether and under what conditions EFHIA can be effective [1, 23, 55].

**Figure 1:**
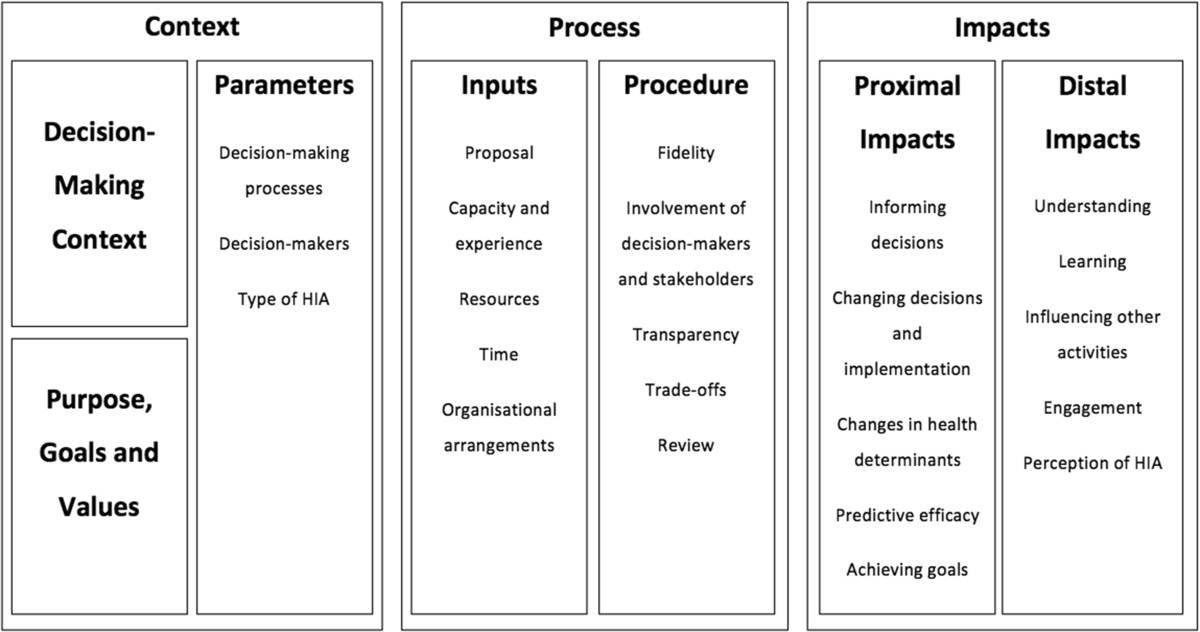
**Original conceptual framework for evaluating the impact and effectiveness of health impact assessment.** Source: Harris & Harris-Roxas 2013 [74].

What is meant by effectiveness in relation to HIA, and impact assessment in general, remains difficult to assess. At one level the effectiveness of HIA can be said to be measured on the basis of whether an HIA’s recommendations were accepted, adopted and implemented. At another level it can be said to require a much broader conceptualisation of effectiveness that encompasses direct and indirect, immediate and longer term impacts [74]. The tension between these approaches to thinking about HIA’s effectiveness led the authors to build upon previous approaches to evaluating HIA [65, 68, 72, 76–78] to develop a conceptual framework that encompasses a broad range of contextual, process and potential impacts factors (see Figure 1).

The process for developing this framework [74] highlighted that measures of effectiveness that focus simply on the extent to which an HIA’s recommendations are implemented misses many of the most important and valued impacts stemming from an HIA. These include factors such as changes to ways of working, learning, and engagement and collaboration. This view is consistent with the discussion and conclusions of other research on the effectiveness of HIA [3, 69, 76, 78].

This paper reports on the first known study to draw on several EFHIA case studies in order to identify EFHIAs’ potential impacts on decision-making and implementation in health service planning. It tests the conceptual framework for evaluating the impact and effectiveness of HIA (see Figure 1) [74] to see if it applies to EFHIA and to identify what modifications may be required, as well as identifying factors that may promote and impair the impacts of EFHIAs on decision-making and implementation.

### The context for this study

The three EFHIA cases in this study were undertaken in New South Wales (NSW), an Australian state with 7.3 million residents, the majority of whom live in its capital Sydney. In Australia health service delivery is largely the responsibility of state and territory governments, with the Federal government funding a range of primary health, disability and aged care services. Two of the EFHIAs were conducted within the NSW Ministry of Health (at that time called the NSW Department of Health) and one was conducted in an Area Health Service, which are semi-autonomous regional health organisations overseen by the Minister of Health.

The use of EFHIA was pioneered in Australia, Wales and other parts of the United Kingdom [15, 16, 27, 38, 50, 55, 79] and has subsequently been modified and adapted for use in different countries and contexts [22, 23, 28, 35]. It has evolved into a specific model of practice in NSW, and has tended to be conducted as rapid appraisals on health sector proposals [55]. In all three cases in this study the EFHIAs were conducted as rapid assessments and involved an integrated appraisal step (combined identification and assessment steps) [10, 15]. The case studies ranged in duration from the shortest taking six days to conduct, through to the longest taking almost twelve months to complete (though this was due to delays within the process, the EFHIA still followed a rapid structure [10]).

All three EFHIAs were undertaken during a period of considerable change for the health services in Australia, some initiated through a series of NSW State Government reforms [80] and some brought about by changes in Federal Government health funding arrangements [81]. These had direct implications for the organisations and programs in each of these EFHIA case studies by both creating and impairing opportunities for change and innovation [82]. Though this context of organisational change had an impact on the EFHIAs and the implementation of their recommendations, in many ways periods of health system reform is becoming a normal, ongoing state for health systems in most developed countries [83, 84]. Public health system expenditure and health workforce challenges, coupled with broader financial and economic crises, have led to series of reforms across many countries [85]. As such even though these EFHIAs were undertaken within a period of changes to health service planning, the findings of this study will still have relevance to other contexts.

### Research aims and questions

This paper reports on research that aimed to investigate whether EFHIA could improve the development and implementation of plans within the health sector; which changes occurred as a result of conducting and implanting the recommendations of EFHIAs; and whether EFHIAs are effective and under what circumstances. The research questions included:

1. What are the impacts of EFHIAs conducted on health sector plans?
2. How does EFHIA improve the consideration of equity in health planning?
3. What changes to the conceptual framework [74] are required to evaluate at the impact and effectiveness of EFHIAs, if any?

## Methods

### Study methodology

This study was informed by an interpretive description research paradigm. This approach emphasises an in-depth and nuanced contextual description that draws heavily on interpretation and experience in order to understand practice issues [86, 87]. The overarching methodology for this study was retrospective case study of three completed EFHIAs. Yin’s approach to case studies [88] was followed because it facilitates explanation of the complex causal links in real-life interventions, in this study EFHIAs; description of the real-life context in which the intervention has occurred, in this context the NSW health system; description of the intervention itself, i.e. how the EFHIAs were conducted; and an exploration of those situations in which the intervention being evaluated has no clear set of outcomes, i.e. the broad range of potential changes that might or might not be attributed to the EFHIAs [88].

The cases were identified purposively [89], which involved selecting cases to “illuminate, by juxtaposition, those processes and relations that routinely come into play, thereby enabling ‘the exception to prove the rule’” [90]. Purposive sampling is most useful when one needs to study specific organisational or decision-making contexts with knowledgeable experts involved, as was the case in this study [91]. Cases had to be:

- Rapid EFHIAs that had been completed between 2006 and 2008;
- Conducted on health service plans;
- Conducted in NSW (the state where the authors are situated, have the strongest connections to health services, and to ensure broad similarity in the organisational context between cases);
- A mixture of centralised (NSW Ministry of Health) and localised plans (HNEAHS); and
- A mixture of effectiveness (EFHIAs that were regarded as having changed the health service plan and those that weren’t).

Four potential cases were identified. Three were included in this study due to resource constraints. The three cases included were selected because they represented the broadest range across the criteria above. The fourth case was excluded because it was conducted in a similar setting as Case Study 1 described below, on a similar type of proposal and was described as being similarly effective.

The background of each of the individual EFHIA case studies and their subsequent impacts on decision-making and implementation are outlined in Boxes 1–3 in the results section. The findings across cases and the implications for the conceptual framework [74] are also presented in the results section.

### Data collection

The qualitative data collection methods are outlined in considerable detail in Additional file 1 and Additional file 2 using the CORE-Q criteria for reporting qualitative research [92] and the RATS qualitative research review guidelines [93]. Fourteen participants were identified purposively to ensure a mixture of people responsible for developing the health service plan, those involved in the EFHIA, and those responsible for acting on its recommendations (several interviewees fell into multiple categories, see Table 1).

**Table 1:** **Characteristics of interviews and documents included in the analysis**

Case study	Total number interviewed	Number interviewed involved in development of original plan*	Number interviewed involved in EFHIA process*	Number interviewed involved in implementing the EFHIA’s recommendations*	Documents included in analysis
Case Study 1: The Good for Kids, Good for Life EFHIA	5	3	4	2	3 (original program plan, EFHIA report, implementation plan)
Case Study 2: The New South Wales Australian Better Health Initiative Implementation Plan EFHIA	5	3	3	3	2 (draft implementation plan, EFHIA report)
Case Study 3: NSW Sexually Transmissible Infections Strategy EFHIA	4	1	3	2	2 (draft strategy, EFHIA report)
Total Across Cases	14	7	10	7	7

*N.B. Several interviewees fit into multiple categories so the sum between columns exceeds the total number interviewed.

Participants were approached to be interviewed by email (11) or phone (3) and all potential participants who were approached agreed to be interviewed (100% participation rate). A written information sheet about the study was provided to each participant along with details of ethics approval. Written consent was obtained from participants. Semi-structured interviews followed a guide (see Table 2) and where possible documents relating to the original plan, the EFHIA and subsequent implementation documentation were obtained (see Table 1). Each interviewee was asked whether there were any non-confidential documents relating to the EFHIA process that could be included in the document analysis. In two of the three cases more than one interviewee identified and shared the same documents with the study team.

**Table 2:** **Semi-structured interview guide**

1)	Tell me in your own words how the EFHIA was undertaken (Prompt: And then what happened?)
2)	What changed as a result of doing the EFHIA?
3)	Was the EFHIA a success? Why?
4)	In general, what would make an EFHIA successful?

### Analysis

Data from both the interviews and the documents were imported into NVivo qualitative data analysis software [94] and then coded using the conceptual framework as categories (Figure 1). The data were subsequently free coded [95] to establish:

- if there were distinct concepts in the data that didn’t to fit into the conceptual framework;
- if there were concepts in the conceptual framework that weren’t found in the data; and
- what topics were discussed in uncoded or sparsely coded portions of the data (portions of the interviews and documents with only one code or no coding after the initial coding pass).

Though this was not a grounded theory study, the constant comparative method informed the approach to coding by identifying any differences between respondents based on their role in the EFHIAs, and differences between the three EFHIAs (see Table 1 for an overview) [96].

The interview data was broadly similar in format as the interviews were structured around a semi-structured interview guide (see Table 2). The documents took quite differing forms; some were detailed textual descriptions whereas other documents were tables describing activities. These data was coded using the same process and approach as for the interview data but with specific reference to considering what information that might be expected was excluded as well as included in the documents. The importance of this approach is emphasised in the literature on document analysis [97].

Validity enhancement activities were undertaken through a “coding workshop” and checking coding with other two other researchers. A thirty-minute coding workshop was undertaken with six social researchers looking at two one-page excerpts from two separate interviews. The data was discussed along with what major themes were present. The workshop also discussed how these data might be coded against the conceptual framework [98, 99]. A sample of the data (three interviews, the longest one from each case) was coded by two other researchers: one with a familiarity with HIA but not the cases in question; and another with no background in HIA but with familiarity with health service planning. The range of codes identified was similar and a limited number of differences in coding were resolved through discussion. Both these activities were undertaken to ensure broad similarity and agreement on coding and that major emergent themes were identified.

The overall analytic approach and validity enhancement measures adopted are described in detail in Additional files 1 and 2.

### Ethics approval

Ethics approval for this research was obtained from the University of New South Wales’ Human Research Ethics Advisory Panel I: Social and Health Research (9_08_121).

## Results

Results from this study are presented in two sections – a section describing each of the three case studies and their impacts on decision-making and implementation, and then a section describing results across cases. The categories and sub-categories developed through coding the qualitative data (coding nodes) are described in Additional file 3[95, 100].

### Case descriptions

The EFHIAs each had differing degrees of perceived effectiveness. A description of each case, its context, EFHIA process and subsequent impacts are included below. Each case description outlines the factors have played a role in enhancing or limiting the impact of the EFHIAs on decision-making and implementation.

#### Case study 1: The Good for Kids, Good for Life EFHIA

*Good for Kids, Good for Life* was a four-year population level, multi-pronged childhood obesity initiative in Hunter New England Area Health Service (HNEAHS). The initiative received $7.5 million in funding from the NSW Department of Health and the local Area Health Service. It was a significant program with school, child-care, health service and social marketing components. A rapid EFHIA was conducted between 2007 and 2008 to assess potential differential health impacts on Aboriginal children and young people to ensure the program did not exacerbate existing inequalities between Aboriginal and non-Aboriginal children.

The EFHIA drew on information from over 50 Aboriginal community consultations that were conducted in 30 Aboriginal communities across HNEAHS; population profiles of Aboriginal communities across HNEAHS; and a two-day appraisal workshop with experts and key stakeholders. The EFHIA sought to identify factors that would facilitate or hinder Aboriginal children’s capacity to participate in the *Good for Kids, Good for Life* program, to eat healthily and to be active. It did not seek to address other potential inequities that could arise from the initiative in terms of age, gender, socioeconomic status or location, except insofar as these were considerations within Aboriginal population sub-groups.

The EFHIA recommended over 80 modifications to the program focused on providing education on nutrition, working through schools, addressing transportation barriers to healthy eating and physical activity, providing weight management advice and ensuring that participation in the program did not incur any direct costs for children or families. The EFHIA steering group also developed substantial guidance based on ten major themes on how the program could best work with Aboriginal communities, including ongoing consultation, use of culturally appropriate materials and working with well-known Aboriginal role models. The EFHIA recommended incorporating additional settings be added to the program’s settings-based approaches (e.g. Aboriginal Community Controlled Health Organisations) [101] and amending policy templates and resources to improve cultural appropriateness.

All the EFHIA’s recommendations were implemented and documented in revisions to the *Good for Kids, Good for Life* program plan, a detailed plan that implementation. The plan was regularly formally reviewed as part of the program’s implementation, which involved managers responsible for different aspects of the program meeting to report on progress against every item on the implementation plan. The EFHIA was described by those impacts as having an impact on all aspects of the program because all recommendations were adopted and built into the program’s planning framework. The EFHIA was also described as influencing work practices in other programs and parts of the health service, in particular how population health activities sought to consult and involve Aboriginal communities. The EFHIA was recognised more broadly by receiving the 2008 New South Wales Health Minister’s Award for Aboriginal Health. The NSW Minister for Aboriginal Affairs, Paul Lynch, commended the EFHIA, saying “this project brings together a variety of agencies, community groups and industry to provide practical information… to make it easier for Aboriginal children to be active and eat well” [102].

Factors that facilitated the impact of the EFHIA on decision-making included:

- A high level of involvement of the Good for Kids program management in the EFHIA; and
- The commitment of the organisation to act on the findings of the EFHIA.

Factors that impaired the impact of the EFHIA included:

- Many aspects of the broader *Good for Kids, Good for Life* program had already begun implementation before the EFHIA was completed, potentially limiting the nature of changes and modifications that could be made to the program; and
- The HIA’s focus on Aboriginal children and family limited the extent to which other potential health equity impacts could be addressed, though several interviewees suggested that if the program worked to address the needs and concerns of Aboriginal people, the needs of other disadvantaged groups would be indirectly addressed as well.

#### Case study 2: The New South Wales Australian Better Health Initiative Implementation Plan EFHIA

The Australian Better Health Initiative (ABHI) Implementation Plan was developed as part of a Council of Australian Governments (COAG) package aimed at achieving better health for all Australians through a focus on the prevention and early detection of chronic disease [103]. There had been an increasing recognition by both state and federal governments that there was a need for sustained investment in prevention in order to address issues such as healthy ageing, workforce health and productivity, increases in rates of chronic disease and risk factors associated with chronic disease, and widening health inequities. Combined, these issues had the potential to undermine the sustainability of the overall health system by increasing the burden on acute care services. As part of its response to the overall ABHI plan, NSW Treasury allocated $20.1 million in new funding to be used over four years to enhance programs for promoting healthy lifestyles and supporting healthy lifestyle and risk factor modification. This represented a substantial increase in funding for preventive health in the state, and importantly it was a new pool of funding.

The NSW Ministry of Health (at that time the Department of Health) developed a series of initiatives within a very short timeframe, in order to respond to the deadlines imposed by the COAG planning process. These draft initiatives were included within the Implementation Plan and circulated to key stakeholders for comment, which led to the suggestion that an EFHIA could be undertaken on the proposals. The Ministry of Health agreed to the EFHIA provided (i) it could be done within 4 working days as the final document needed to go to the Minister of Health three days after this deadline, (ii) did not suggest new strategies but made recommendations on how existing strategies could be strengthened or modified, and (iii) did not recommend changes in funding levels. Issues related to Aboriginal health were excluded from the EFHIA as these were being covered through a separate Aboriginal Health Impact Assessment process [104].

The EFHIA was scoped to look at two components within the ABHI implementation plan (promoting healthy lifestyles and supporting lifestyle and risk modification), in order to respond within the timeframes available. The EFHIA drew on a rapid review of the literature, a one-day workshop with seven key stakeholders from government and universities in NSW and Victoria. The EFHIA recommended a series of changes to items within the implementation plan. These recommendations were aligned to the existing structure of the implementation plan. For each item within the implementation plan the EFHIA included one page outlining:

1. What is the initiative trying to do?
2. Is there evidence of inequity?
3. Who may be disadvantaged by the initiative?
4. Are there likely to be unanticipated impacts?
5. What are the key recommendations for implementation?

The extent to which the EFHIA’s recommendations were implemented remains contested and unclear. Some of the people interviewed indicated that there were clear changes to planning and implementation that could be attributed to the EFHIA. Others reported that these changes would have been made anyway as part of routine planning and program development processes, and that many of the changes to implementation could not be attributed to the EFHIA but to other contextual factors. It was acknowledged by all interviewed that the implementation plan had changed, but there were different views about what these changes should be attributed to.

Factors that facilitated the impact of the EFHIA on decision-making included:

- A willingness and openness by the Ministry to have the draft implementation plan reviewed; and
- Adapting the EFHIA process to respond to time pressures.

Factors that impaired the impact of the EFHIA included:

- The limited number of people directly involved in the EFHIA process and that these people did not directly include the people responsible for implementing the EFHIA’s recommendations, due to a number of timing and decision-making contextual factors; and
- Individual responses influenced how the EFHIA’s recommendations were received, in particular the extent to which the EFHIA was perceived to be unduly critical.

The process this EFHIA followed and its impacts on decision-making and implementation have been described in considerable detail in a paper in *the International Journal for Equity in Health*[55].

#### Case study 3: NSW Sexually Transmissible Infections Strategy EFHIA

The *NSW Sexually Transmissible Infections Strategy 2006–2009* was the first STI strategy to be developed in NSW. The strategy identified a number of priority groups: Aboriginal people; gay and other homosexually active men; young people; sex workers; people with HIV/AIDS; people who inject drugs; and heterosexuals with recent partner change. These priority populations were identified based on epidemiological evidence about groups with higher rates of STIs, groups with relatively lower rates of STIs where the rate has been increasing, and groups identified as having relatively higher numbers of sexual partners.

The strategy set out a number of areas for activity, including:

- promoting general STI awareness;
- working with primary health care providers (general practitioners);
- prioritising access to and the focus of publicly funded sexual health clinics to those priority groups described above;
- promoting STI testing;
- improving contact tracing;
- strengthening health promotion programs around sexual health;
- developing the workforce; and
- research and surveillance priorities.

The EFHIA was suggested as an activity within the NSW Health Public Health Officer (PHO) Trainee program, which trains a cohort public health officers within the NSW health system in a broad range of public health skill areas. The strategy was identified by the manager of the PHO Trainee program in conjunction with the manager of the AIDS and Infectious Diseases Branch as being appropriate for an EFHIA. This was because it was undergoing a mid-term review in 2008, which allowed an opportunity for the EFHIA to guide and inform any changes that might be required whilst having a clear and well-structured strategy to assess. The stated objectives of the EFHIA were:

- To create a learning based exercise for the NSW Health Public Health Officer trainees;
- To review the policy and make equity-based recommendations to support the development and implementation of the next strategy; and
- To engage the AIDS and Infectious Diseases Branch within the Centre for Health Protection in the use of EFHIA.

The EFHIA was conducted following a rapid process with three workshops over a two-week period – one for screening and scoping, one for identification and assessment, and a final one for development of recommendations. Between the workshops three of the PHO Trainees undertook a rapid review of the literature and compiled a profile of STI transmission in based on NSW Health data, with a focus on identifying sub-populations with high rates of STIs and new and emerging patterns of infection. The participants numbers varied across the three EFHIA workshops but included a mix of PHO trainees and staff from the Centre for Health Advancement and the AIDS and Infectious Diseases Branch within the Ministry of Health. The PHO Trainees had all previously received 4 hours introductory training in HIA. Technical procedural support for the EFHIA was provided by a lecturer from the University of New South Wales with a background in health impact assessment.

The EFHIA made a number of recommendations, which lead to an increased emphasis on access to services by groups within priority populations, such as Aboriginal communities in regional and rural areas. These resulted in changes to the draft document. The EFHIA also strengthened the Strategy’s emphasis on working with primary health care as the principal mechanism to address issues of access for advice and treatment, as well as identifying people at risk.

Factors that facilitated the impact of the EFHIA on decision-making and implementation included:

- The willingness of the AIDS and Infectious Diseases Branch, who were responsible for revising and implementing the strategy, to have the EFHIA conducted and to participate in the process;
- The availability of PHOs to assist in the EFHIA and their diverse range of skills; and
- A clear, structured proposal to assess in the form of the strategy.

Factors that impaired the impact of the EFHIA included dDiffering perceptions of the purpose of HIA, with some participants regarding it solely as a training exercise with no scope to change the proposal, whereas others regarded it as a legitimate activity with scope to affect change (notably including the AIDS and Infectious Disease Branch, who were responsible for implementing the proposal). The EFHIA was conducted to inform a a mid-term review and as such there was not as much scope to alter fundamental aspects of the Strategy as there might have been if it was a newly developed strategy, though this needs to be balanced against the greater detail that was available to inform the assessment.

There were challenges reconciling conceptual differences between an equity analysis based on potential dimensions of within-population inequity (the EFHIA looked at differences in terms of age, gender, socioeconomic position, location, existing levels of health and disability, sexuality, etc.) and a strategy that was developed with close attention to empirical data on the prevalence and transmission of STIs within specific populations (the STI strategy was developed to target specific priority populations, as well as strengthening health service links). This involved re-examining knowledge and assumptions about STI priority populations, as well as considering within-population differential impacts that could arise as a result of the policy.

### Results across cases

The conceptual framework for evaluating the impact and effectiveness of HIA (see Figure 1) was used to structure the presentation of results across the cases [74]. This framework has been used elsewhere to frame analysis and discussion of HIA case studies [105] and looks at a broad range of context, process and impact factors that influence, and are influenced by, HIAs. This structure was also used because one of the aims of this paper was to examine what changes to the conceptual framework were required when evaluating the impact and effectiveness of EFHIA, as distinct from HIA.

## Context

### Decision making context

At a broad level there was a lot of similarity between the three case studies’ decision-making context, which reflects the purposive nature of the case selection (see Methods). All cases were EFHIAs conducted on NSW health sector plans within a two-year period. Two of the case studies were from within the central Ministry of Health office; one was conducted within a local health district.

There was broad consistency in the approach to health service planning across all cases, which involved developing draft plans; consulting with a number of internal and external stakeholder groups; and reviewing related guidance, evidence and best practice. All three cases took place within a period of significant organisational change in the NSW health system, as discussed in the background section.

### Purpose, goals and values

#### Purpose

Agreement or disagreement about the purpose of the EFHIAs was a significant factor that affected how the EFHIAs were conducted and its recommendations received, and the issue was relevant in all cases. Only one of the EFHIA reports stated its purpose clearly and unambiguously. Interviews highlighted that there was considerable variation about the NSW STI Strategy EFHIA’s perceived purpose, specifically about whether its main purpose was to be a training activity or to inform the development and implementation of the Strategy. There was also some variation between interviewees about the perceived purpose of the ABHI Implementation Plan EFHIA:

“[The EFHIA was] a kind of a training opportunity for the Public Health Officer trainees in the first instance. So that was kind of its primary purpose and then it had a happy spin off of being something that could usefully inform our work.”

NSW STI Strategy EFHIA interviewee

“There are quite dichotomous views about what people believe about HIAs. Some people believe there is a place [for HIAs], blah, blah, blah and they’re fantastic. Other people believe [these issues are addressed as] part of a good planning process, and there’s some there are in between those two.

ABHI Implementation Plan EFHIA interviewee

#### Goals

The goals of the EFHIA were not clearly stated in the documentation for two of the EFHIAs, though the goals of the original plans were articulated in all three cases. Goals were implied rather than stated in the interviews.

#### Values

There was explicit reference to equity in all three cases, mostly through the language used in the interviews. This may be unsurprising given they were all EFHIAs and equity is an explicit value described in the title of the process. There were very few instances of the explicit description of values in the documents analysed. There was considerable overlap in the way the purpose, goals and values of the EFHIAs were discussed in the interviews and documentation. A number of interviewees suggested that the EFHIAs may have had an impact on participants’ values, but also identified this as an area of conflict or change that failed to eventuate.

It would have been a success if that was the case, you know, those sort of what we call a, you know, a more indirect impact around values, changes and stuff like that. That I would consider that as a success.

NSW STI Strategy EFHIA interviewee

### Parameters

#### Decision-making processes

There was recognition in almost all interviews that the EFHIAs took place within broader decision-making processes, such as funding agreements between organisations. The documents described these decision-making processes well, as they provided clear boundaries for the scope of the EFHIAs. Several interviewees described this as a factor that facilitated the EFHIA by making clear what decisions had already been made and which were still possible to influence or change.

So we obviously need to be really clear from a Department point of view about what you could comment on, and what you couldn’t comment on.

ABHI Implementation Plan EFHIA interviewee

#### Decision-makers

Decision-makers were consistently identified as a factor that set the boundaries in the EFHIAs before they had commenced. The extent to which the people who were in a position to act on the recommendations were receptive to an EFHIA being conducted in the first place was described as a significant factor that either helped or hindered all three EFHIAs, and seemed to vary between them.

The EFHIA happened after we circulated the plan for comment… [The EFHIA wasn’t my idea, someone else] was pushing for the HIA.

ABHI Implementation Plan EFHIA interviewee

The HIA process was actually um… really useful for trying to, for demonstrating that we, as a project, were committed to, to listening and making changes.

Good for Kids, Good for Life EFHIA interviewee

#### Type of HIA

All three EFHIAs that were conducted rapidly, as that was one of the case selection criteria. The interviews confirmed that the desire to address equity well informed the very earliest decisions about whether to conduct the EFHIAs. All three EFHIAs were described as rapid and were intended to be conducted within short timeframes. The actual duration of the process varied markedly between the EFHIAs, ranging from a week to several months, though the amount of time invested, the approach to data collection and the use of rapid appraisal workshops to synthesise the evidence from multiples sources was quite similar across all three cases.

#### Timing of when the HIA is conducted

A significant parameter that was identified in the interviews, which had previously not been described in the conceptual framework (see Figure 1), was the timing of when the EFHIA was conducted. The extent to which an EFHIA was conducted at the right stage in planning was identified across all three cases as a critical factor that influenced everything that came afterwards, including the process for the EFHIA being conducted but also extending to the extent to which recommendations were appropriate or addressing activities that were amenable to change. Whilst some of the interviewees recognised that there was value in having enough detail in the proposals to assess, most expressed concern that too many of the higher-level decisions about what the main features of the plans had already been made.

I would have said, “This is not a good thing to be doing an HIA on. It’s too complete, it’s too difficult to change. I understand that the idea is that you might be able to influence the next one, but it’s not an appropriate thing to be doing it on”.

NSW STI Strategy EFHIA interviewee

We actually started doing it after the project had already been commenced. But I think that was the difficulty. Because it was so hard to go back. And it should be something that’s done prior, whereas this wasn’t done prior.

Good for Kids, Good for Life EFHIA interviewee

## Process

### Inputs

#### Proposal

The timeframes for developing the initial plans that the EFHIAs assessed varied markedly, ranging from 2–3 weeks (the ABHI EFHIA) through to more than a year (Good for Kids, Good for Life EFHIA). Despite this all three cases had clear, well-described proposals to assess. The ABHI Implementation Plan EFHIA in particular had a clear proposal but also had a clear brief for the assessment team that set out the four components of the Implementation Plan that the Department agreed to being examined through the EFHIA.

I’m also not convinced that a rapid HIA on a document with only four pieces of the jigsaw puzzle was a good idea, would I do it for the next bit of the Implementation plan, I don’t know. I like the idea of a rapid HIA, because then presumably it fits into all our timeframes, which are often unrealistically ridiculous… So one way I like the idea of that, I don’t know.

ABHI Implementation Plan EFHIA interviewee

#### Capacity and experience

The experience, individual capacity and organisational capacity of those involved in the EFHIAs were described as a facilitating factors in all but two interviews. In all three EFHIAs the participation of people with experience in conducting EFHIAs, expertise in the proposal area and knowledge about related health equity issues was described as helping the EFHIA process.

I think as an experienced person when they try, you know instinctively, early on and try to see where things can go wrong. I could see the potential for absolute disaster going down a quite a sophisticated approach to the [assessment] matrix, so we used [an appraisal workshop]. Um, and ah, I think the EFHIA questions capture, they capture it, they capture the system.

NSW STI Strategy EFHIA interviewee

Another aspect of experience and capacity that was identified in the interviews was the involvement of the people who had developed the proposal being assessed. This involvement took different forms in each of the EFHIAs, largely due to competing time pressures. This extent of involvement assisted the EFHIA process but was also described as altering the way recommendations were framed and enhancing the impact of the EFHIA on decision-making and implementation. The EFHIA with the highest level of involvement of those responsible for developing the proposal was the Good for Kids, Good for Life EFHIA. Four interviewees for this EFHIA described this high level of involvement as enhancing the process and impact of the EFHIA.

Yeah, yeah and once the recommendations were sort of offered and strategies presented back and negotiation around them to give them what we wanted. But they became a part of the program plan, so yeah that’s sort of our main governing document. So if it’s in the programme plan, they had to report on it to sort of their manager and then up to the program advisory committee.

Good for Kids, Good for Life EFHIA interviewee

#### Resources

Resources devoted to the EFHIAs took several forms including financial support, providing venues and logistical support for the appraisal workshops, and the provision of EFHIA technical and advice and support from the University of New South Wales. The most important resource discussed in the interviews however was the time of those involved in the EFHIA, most of which was paid by their employers. Two participants in the Good for Kids, Good for Life EFHIA were the only people in all three EFHIAs who were not participating as part of their paid employment.

#### Time

The time available to conduct the EFHIA was recognised as a significant factor that affected how the EFHIA was conducted. All three EFHIAs were rapid in nature, largely due to time pressures imposed by external decision-making processes. For example the bulk of the ABHI Implementation Plan EFHIA was completed in five working days in order to meet timeframes imposed by Council of Australian Governments (COAG) processes.

Yeah, the turnaround was ridiculous, and I certainly appreciate from our point of view it was going to be ridiculous, but even more so from the people who were doing [the EFHIA], it was going to be ridiculous. We were given a very tight timeframe of when things needed to be approved by the Department, and ah, that was tied up to some extent in the COAG process.

ABHI Implementation Plan EFHIA interviewee

The Good for Kids, Good for Life EFHIA also had time pressures on it, given the program was being implemented at the same time that the EFHIA started. However instead of compressing the time available several people involved in developing and implementing the plan recognised there was a need to invest in understanding the EFHIA process and building trust with members of the EFHIA advisory group. Though this explanation and trust-building took some time, the EFHIA itself remained rapid in nature.

We had an advisory group in place um that advised on a range of things that relate to how we interact and operate with Aboriginal communities in the region. And we needed to sell this idea to them. And that was a bit of work. And it’s, ah, it’s a, the, the process they needed to understand and that took a while. But also they needed to be able to see what benefits it was going to bring in the long term and why it was worthwhile participating in this process. And that, that was hard work.

Good for Kids, Good for Life EFHIA interviewee

#### Organisational arrangements

Existing organisational arrangements significantly affected the process across the three EFHIAs. Both the NSW STI Strategy EFHIA and the ABHI Implementation Plan EFHIA mostly involved stakeholders within the NSW health system. This provided a clear context for why the proposals were important and provided an impetus and a degree of assumed agreement about their participation in the EFHIA. It also meant there was some degree of recognition of the importance of health equity and the NSW health system’s commitment to it as a value informing health service planning and delivery [54].

The Good for Kids, Good for Life EFHIA involved a greater number of external stakeholders including Aboriginal community controlled health services, the state government departments for education and community services, Aboriginal health workers within the health system, and community representatives. They had to invest much more time explaining the proposal to stakeholders and why their participation was important, compared to the other two EFHIAs in this study.

#### Individual agency

Several interviewees emphasised the difficulties in engaging in a process that was not their choice to undertake or which they described as being thrust upon them. This lack of control or agency was often described when they were explaining why the EFHIA had limited impacts or wasn’t well aligned with decision-making processes. Conversely, in the interviews where people said they played a role in initiating or voluntarily participating in the EFHIA they described this as leading more easily to implementing the EFHIA’s recommendations, illustrating both aspects of the role individual agency played in the EFHIAs.

Okay, when HIA came up, we’d only heard briefly about it. I’d heard about it. I’d never worked on a HIA before in that context… One of the things that can be a bit daunting too, and I’m going to make a sort of assumption statement now, one of the things that can be quite daunting is someone from the [university] comes in and says, ‘you beaut, great, fantastic tool to use’. If you haven’t had experiences with that before, often you’ll think, ‘well, yeah, okay lets run with it’.

Good for Kids, Good for Life EFHIA intervieweeThis item was not in the original conceptual framework (see Figure 1) but arose consistently in interviews as a distinct factor that influenced how the EFHIA was conducted, how its recommendations were received, and the extent to which it has an impact on subsequent decision-making and activities.

### Procedure

#### Fidelity

In all three EFHIAs there was a high degree of adherence to established guidance on the procedural aspects of EFHIA. The only difference to the process described in some HIA guidance was that all three involved an integrated appraisal step, rather than separating out identification and assessment [9, 10]. This meant that information on the likelihood and magnitude of potential impacts was assessed as it was gathered, using a collaborative group process [27, 79], rather than reporting all potential impacts and then assessing them as separate steps. This was described as being due to the rapid nature of the EFHIAs and does mirror the process described in the original EFHIA Framework [15].

#### Involvement of decision-makers and stakeholders

There was marked variation in the level of involvement of decision-makers and stakeholders between the EFHIAs. In the Good for Kids, Good for Life EFHIA people who had the capacity to alter the implementation of the program were actively engaged throughout the process. In the cases of the NSW STI Strategy EFHIA and the ABHI Implementation Plan EFHIA the people responsible for implementing and overseeing the development of the plan were not able to be actively involved in all aspects of the EFHIA process, in both cases due to competing time pressures and other activities associated with the plans being assessed.

This was identified in the interviews as a critical factor that has the ability to assist or impede subsequent impacts on decision-making and implementation.

Well, I think one of the things seems to be to have in the room, during the assessment phase, people who can influence the outcome, because a lot gets lost in translation, and it’s actually the discussions around why you’ve come up with the recommendations which are important, and that if you’re not involved in those discussions, it’s not always obvious how you went from Point A to Point B. So I think that’s important, but probably unrealistic in many situations, but as much as you can, to get people who can influence the implementation involved, I think, because in a way, it was about improving the quality of the document, it was actually quite important to be able to debate some of the issues.

ABHI Implementation Plan EFHIA interviewee

#### Transparency

All three EFHIAs documented and reported on the process they followed well, and the description of the process followed in the interviews was consistent with that described in the EFHIA reports.

#### Trade-offs and review

These factors were included in the original conceptual framework (see Figure 1) but weren’t found in either the interview or document analysis data in this study.

## Impacts

### Proximal impacts

#### Informing decisions

All three EFHIAs were described as informing the thinking about the proposals assessed and informing subsequent decisions, though the extent and nature of that change varied a lot. The extent to which they informed decisions seemed to be associated with the level of involvement of those responsible for implementing the plans in the EFHIA process.

If [the EFHIA] had been built in earlier, I would have had more ownership of it. And certainly if anyone above me had built it in [to the planning process], I would have felt a greater sense of responsibility to act… So I think making sure the people at the right level are involved at the right, at an early stage.

NSW STI Strategy EFHIA interviewee

Although almost all interviewees described the EFHIAs as informing subsequent decision-making to some extent, this was not necessarily described as leading to changes to decisions and implementation.

#### Changing decisions and implementation

The extent to which the three EFHIAs in this study influenced subsequent decision-making and implementation varied markedly, even when described by interviewees involved in the same EFHIA. Only one of the documents available to be analysed had been formally revised following the EFHIA (The Good for Kids, Good for Life implementation plan). This document showed that all the recommendations in the report were clearly incorporated into the implementation plan. This process was described in interviews as involving a degree of modification and negotiation but also emphasised that once recommendations were contained in the implementation plan they would be monitored for progress and reported against.

But the, the beauty of it was that [the EFHIA] wasn’t my responsibility any more. It was sort of becoming embedded across [the program]. Yeah, yeah and once the recommendations were sort of offered and strategies presented back and negotiation around them to give them what we wanted. But they became a part of the program plan.

Good for Kids, Good for Life EFHIA interviewee

It is more difficult to point to concrete changes arising from the other two EFHIAs in this study. Interviewees disagreed about the extent of change that could be attributed to the EFHIAs. Both plans undertook substantial changes in response to broader changes to the NSW health system following the EFHIAs, which limited the extent to which subsequent changes can be attributed to the EFHIAs.

The positive thing that came out of it for me was that ah we heard some things had been changed. The difficulty was, and um, was that we had no idea what had been changed and we had no access to the documentation. And we had no access to the decision making around it.

ABHI Implementation Plan EFHIA interviewee

To be honest, I’m not sure that much else came out of it. I think, you know, given how difficult it was, I think just the fact that maybe some people might consider using health impact assessment and that we may have influenced the Strategy are not bad outcomes.

NSW STI Strategy EFHIA interviewee

#### Changes in health determinants

Three interviewees described addressing the determinants of health as an important intent underpinning the use of EFHIA, though they were not able to identify any changes to specific determinants arising from the EFHIAs they participated in. Two of the documents analysed made explicit mention of the determinants of health.

#### Predictive efficacy and achieving goals

These impacts were included in the original conceptual framework (see Figure 1) but were not found in either the interviews or document analysis. Predictive efficacy refers to the extent to which predicted impacts eventuated and achieving goals refers to the extent to which the stated goals of the assessment were met. Both these factors seem to have been of limited relevance in the EFHIAs in this study, though this may be due to the study’s setting, i.e. rapid EFHIAs being conducted voluntarily rather than to meet a regulatory requirement.

### Distal impacts

#### Understanding

The EFHIAs were all described as leading to better understandings of how other agencies worked, and the pressures and concerns that informed health service planning. They also led to understanding of ways of working in partnership with other stakeholders.

[The EFHIA] made them think about and what our [Aboriginal communities’] way of doing business is. Don’t like this approach, the major consultation processes that needed to be undertaken before it actually was, before it was to be done. And that’s my recollection. I think I actually thought [the proposal] had some good points to it. I think it was a valuable process but it would be more valuable if it had been thinking about this stuff when they planned it.

Good for Kids, Good for Life EFHIA interviewee

The EFHIAs were also described as leading to better understandings of planning processes and how the plans were originally developed, though this view was contested in some cases.

Yeah, I think in hindsight, I would want to know more about why [we would] would want to do one, and what they hoped to get out of it, and I would want [people undertaking the EFHIA] to know more about what we would hope to get out of it, so that those misunderstandings or miscommunications didn’t happen in the process.

ABHI Implementation Plan EFHIA interviewee

#### Understanding of health equity specifically

Understanding of health equity and the determinants of health inequalities was highlighted as a major impact of all three EFHIAs. This was described as better understanding of the (i) potential health inequities that could arise or be exacerbated as a result of the type of proposal being assessed, and (ii) the distribution of potential impacts amongst population sub-groups based on different approaches to disaggregation (age, gender, socioeconomic status, location, etc.).

This change was likely to be due to the explicit focus on health equity in all EFHIAs. The extent to which understandings of equity changed as a result of the EFHIA varied between the three case studies, and even between interviewees within each one. The level of involvement in the EFHIA process (being the person responsible for undertaking the EFHIA, participating in the assessment/appraisal step, etc.) seemed to be closely associated with the extent of improved understandings of health equity, though this was not universal amongst the interviewees.

Understanding of health equity in the context of health service planning was also recognised by interviewees as not being straightforward:

I think there is something conceptually difficult about saying, “Okay, well you’ve identified gay men and drug users but then, who among those groups and more, you know that sort of… how do you prioritise… I mean, you know, how do you, and clearly with gay men you could, you could prioritise young gay men or do you could prioritise homeless young gay men… It really adds a layer of complexity and it makes it quite hard to conceptualise what you’re trying to achieve.

NSW STI Strategy EFHIA interviewee

I think from my own learning, one of the things we learned, I learnt, was that we overlook gender as one of the dimensions or differential impacts that, throughout the document, particularly things referring to adults, they really treated men and women as if they’re the same thing, and we know that their participation and their engagement’s very different, but we don’t necessarily articulate that… That was an unexpected finding for us, is how easy it is to overlook gender.

ABHI Implementation Plan EFHIA interviewee

This item was not in the original conceptual framework but arose consistently in the interviews and documents reviewed. It was described separately and using different language than was used for other forms of understanding, such as understandings of the determinants of health or understanding how other agencies worked.

#### Learning

The rapid nature of the EFHIAs was recognised by interviewees as responding to the decision-making context but that this may also have impaired the extent to which learning could take place. The nature of learning that was desired and anticipated from the EFHIA also seemed to be varied, with some participants talking about how they hoped the EFHIA would provide technical insights whereas others hoped it would enable people to think about the proposals, and health service planning in general, in a different way. In particular there were differing expectations about the nature and extent of alternatives that might be considered. The EFHIAs were described by four participants as involving a learning new concepts or approaches to addressing health equity concerns.

We were able to enter into some discussions with them about what might be alternatives, so I think that in these sorts of environments, we’ve got an opportunity to influence the implementation. It’s actually really important to have debate, and that’s what I think the EFHIA allowed.

ABHI Implementation Plan EFHIA interviewee

It hasn’t obstructed anyone, in getting them to reflect on their work, really, even if they weren’t, you know, up-skilling in the process of HIA, they probably could have learnt a few things about equity considerations, and how to incorporate that, so I think that might have been a missed opportunity to engage people in the process, probably the rapid nature makes that a little difficult.

ABHI Implementation Plan EFHIA interviewee

#### Influencing other activities

The EFHIAs were described as having impacts on a range of other activities, principally in terms of related planning and implementation issues that crossed over with other parts of health services. This influence on activities could be regarded as both positive and negative. In the ABHI Implementation Plan EFHIA this influence was described as impairing or undermining relationships and potentially limiting future collaboration.

[EFHIAs] can be used to change the way other sectors think about health and equity, like land use plans and that sort of thing, and I don’t think this is something that is going to work with health plans which are already pretty good at health equity. This will probably make me think about how I can use this with local government more though.

ABHI Implementation Plan EFHIA interviewee

Ideally I would like to say that what came out of it was a better relationships I don’t think that happened, but that would have been, in terms of my original thought at the beginning, that was one of the outcomes I had hoped would come out of it.

ABHI Implementation Plan EFHIA interviewee

In the case of the Good for Kids, Good for Life EFHIA it was described by all interviewees as opening up lines of communication within the program and clarifying decision-making and resourcing processes for those involved.

[The EFHIA] suited our purposes for making the programme culturally appropriate, but to do it on its own wouldn’t have done that. We sort of had a sort of a line to three other areas, sort of. So having the consultation or a more comprehensive consultation [that was] being done at the same time. Having, um, Aboriginal people working on the program, so identifying staffing and, also having some sort of resourcing agreement that what came out of it was actually going to be resourced, and like where we can go and do it.

Good for Kids, Good for Life EFHIA interviewee

#### Engagement

The EFHIAs were described by five interviewees as offering more avenues for engagement and participation than would usually be possible in health service planning. This was seen as closely linked to the structured EFHIA process and the degree of collaboration it involved.

Lots of the strategy documents are about, you know, let’s get a bunch of people together and we’ll build a shared understanding and we’ll make a commitment together to move forward with any existing funds, and that can be, be limited.

NSW STI Strategy EFHIA interviewee

#### Perception of HIA

Twelve of the interviewees described the EFHIA process changed their perception and understanding of HIA, and in particular EFHIA, and where it might usefully fit within future planning activities. Even in cases where the EFHIA was described as less successful this change in the perception of HIA was reported.

#### Individual responses

The second coding pass of sparsely coded or uncoded parts of the interviews during the analysis highlighted a number of sections in the interviews where people described how the EFHIA process had changed their perceptions, understandings and relationships at an individual level rather than an organisational one. The language used to describe this was distinct from how the interviewees described organisational responses or how they regarded the EFHIA process. It is important to note however that this individual response as a result of the EFHIA was only reported by six of the interviewees.

I don’t I’ve already said this but in my head that many of them the areas that I probably overlooked the most would [have been] equity related.

Good for Kids, Good for Life EFHIA interviewee

It made me think about some of my kind of thinking.

ABHI Implementation Plan EFHIA interviewee

I wanted to understand the process because it was new to me , but it was hard and it involved a lot of these new ways of thinking about it, and I am an epidemiologist and I just wouldn’t analyse it that way naturally, so I think it changed my sense of how I should think about these problems.

NSW STI Strategy EFHIA participant

This item was not in the original conceptual framework but arose across the three EFHIAs and seems to be related to several other factors in the conceptual framework and is described in greater detail below.

### Other factors influencing the impact of EFHIAs

The other factors that emerged in the analysis as important factors influencing the extent to which EFHIAs appear to have an impact on decision-making and implementation were (i) timing and timeliness and (ii) the interplay between values, agency and learning.The case studies highlighted the need to undertake the EFHIA at the right stage in broader decision-making processes, i.e. early enough to ensure they could usefully inform decision-making. The other aspect of this is timeliness, which was the ability to conduct the EFHIA within the timeframe required or imposed by broader decision-making and implementation processes. There was variation between the case studies in terms of both timing and timeliness and the interviewees did not always describe that timing and timeliness had been well addressed within the EFHIAs. Whilst these factors weren’t the sole predictors of subsequent proximal changes (see Figure 1) they were important ones. This also suggests that timing and timeliness are factors that need to be addressed during the screening and scoping steps for both EFHIAs and HIAs.

The case studies also highlighted the interplay between values, agency and learning as related factors that may facilitate or limit the extent of changes that can occur as a result of EFHIAs. The EFHIAs in this study all involved some examination of potential health inequalities and looking at their distribution, whether these inequalities could be mitigated, and whether they were unfair. In all three EFHIA cases this involved some degree of re-examining organisational and personal values in order to inform whether potential inequalities were unfair and unjust, as well as which potential impacts should be prioritised for action. This necessarily involved revisiting and articulating the values that informed the development of the proposals as well as which values would inform implementation. In this way values played an important role in mediating the potential impacts of the EFHIAs on subsequent decision-making and recommendations.

This examination of values was not necessarily welcomed by all interviewees, particularly in cases where they were not closely involved with the assessment process or in the decision to initiate the EFHIA. They described the EFHIAs as focusing on issues that were not relevant to the decision-making context or not understanding the broader context for the proposal being assessed. The extent to which interviewees were able to express individual agency by initiating the EFHIA or participating in the EFHIA process was also related to whether they saw the EFHIA as successful or not. In every case where the interviewee described the EFHIA as not being a success they were either (i) not involved in collecting and appraising evidence in the assessment process, or (ii) did not play a role in initiating or agreeing to the EFHIA being undertaken.

Individual agency and participation in the EFHIA was linked to values but also appeared to be linked to the nature of learning sought from the EFHIA. Those interviewees who reported being less involved in the process or that it was someone else’s idea often described the EFHIA as inappropriately looking at options and implementation recommendations, whereas they had expected the EFHIA would focus on technical assessment, rather than focusing on implementation, or act as a “learning activity” (a phase used by four interviewees).

I do remember getting it back and going hang on a minute, we gave you really clear parameters about what you’re allowed, or whatever, for want of a better word, ‘to look at’, and it came back saying that. I really believe that it did misrepresent our intention behind it, and why we’d given these parameters around what was fixed and what wasn’t fixed… I think it does misrepresent, and it was quite antagonistic

ABHI Implementation Plan EFHIA interviewee

The HIA was successful, but really just marginally so. The proposal was too developed and worked up to change much, and the equity, the equity issues were not glaringly obvious ones. It was hard for novices, I guess that’s really what we were, hard for us to assess when it was a learning activity.

NSW STI Strategy EFHIA

Conversely those who were actively involved in the EFHIA process through their own choice described gaining new ideas about how to approach the issue the proposal was designed to address and a new appreciation of equity, particularly in relation to the proposal area being assessed.

I think there’s real value in an equity-focused HIA, because I think it does try and make people understand what equity is about. But I do think it’s a very hard concept to grasp, and people look at it, and I think that really happened with this policy, people look at it and they see that you’ve created these priority populations, so therefore you must have considered equity. And trying to get people to dig underneath that, even really quite, you know, educated and intelligent people, can be quite difficult. Because, it’s complicated.

NSW STI Strategy EFHIA

This suggested that there were different understandings about the nature of learning sought from conducting the EFHIAs, ranging from technical to conceptual and even social learning [106, 107]. A shared understanding about the learning desired from an EFHIA, or lack thereof, may have affected its subsequent impact on decision-making and implementation, or even have lead to conflict. This shared understanding about learning also appeared to be linked to the interplay between values, individual agency and learning in these cases. It is important to note that while this interplay affected how the EFHIA was perceived, the effect was not uniform. While most people who had either not been directly involved in the EFHIA or not initiated it described the EFHIA as having fewer impacts, not all did. Even those who were most critical identified a number of positive impacts arising from the EFHIAs, in particular in terms of understandings of equity.

## Discussion

In public health effectiveness is generally regarded as “the positive program outcomes, minus the negative outcomes” [108, 109]. This way of thinking about effectiveness may be less relevant in relation to EFHIA, and HIA in general, because EFHIA is an intervention that attempts to influence attitudes, knowledge, decisions and implementation [74, 110]. The desired outcomes are multifactorial, not universally agreed and potentially contested [1, 53]. This challenges attempts to characterise EFHIAs as simply effective or ineffective, as the results of this study illustrate. Though this discussion section is grounded in the EFHIA case studies included in this study, the issues identified may be relevant to HIA practice in general.

### Perceptions of effectiveness

The case studies showed that some tensions can arise through the HIA process [67]. In the EFHIAs examined these tensions appeared to be linked to three issues. The first of these are that there may be disagreements between stakeholders about the perceived purpose of the EFHIA and what form it should take [53]. Other research the authors have been engaged in suggests that Australian HIAs may emphasise the importance of explicitly stating goals less than HIAs in New Zealand [111], and possibly less than other countries as well.

The second issue was the perception that an EFHIA’s recommendations could have been identified through normal planning and implementation processes and that the EFHIA didn’t necessarily have to be conducted to identify these [67]. In other words, that an EFHIA’s recommendations are “common sense” (a phrase used by one of the interviewees). While some of the recommendations and distal impacts of the three EFHIAs included in this study [74], see Figure 1 could notionally be anticipated through “common sense” analysis, in practice they may have been difficult to anticipate. A similar phenomenon has been noted in other fields such as organisational psychology and management, with information and recommendations being discounted as obvious despite not having been considered in advance [112]. This suggests that what seems like “common sense” may not be obvious in the real world of planning and decision-making. The case studies highlight that there are considerable external pressures on planning activities.

The third issue is the interplay between values, agency and learning. These are all factors affecting the process and impacts of EFHIAs that arise early in the process. This emphasises the need to screen and scope the HIA in some detail and to explicitly define and discuss the purpose of the EFHIA, the values that underpin it and what is hoped to be learnt from it. Recognising individual agency appears to be important in this.

These three issues, about the perceived purpose of EFHIA, the “common sense” nature of HIAs’ recommendations, and values, agency and learning lie at the heart of any appraisal of an HIA’s effectiveness. They are also intrinsically linked to individual perceptions. For these reasons it was often difficult to differentiate between whether an EFHIA *informed* or *changed* decision-making. Checking off an EFHIA’s recommendations against a final implementation plan can indicate some of its proximal impacts see [64] for an example of this, though this will only ever tell part of the story of an HIA’s effectiveness. This highlights the need to collection information on perceptions of effectiveness as a part of any HIA evaluation, an issue that has been under-explored in the literature to date.

### Changes to the conceptual framework

**Figure 2:**
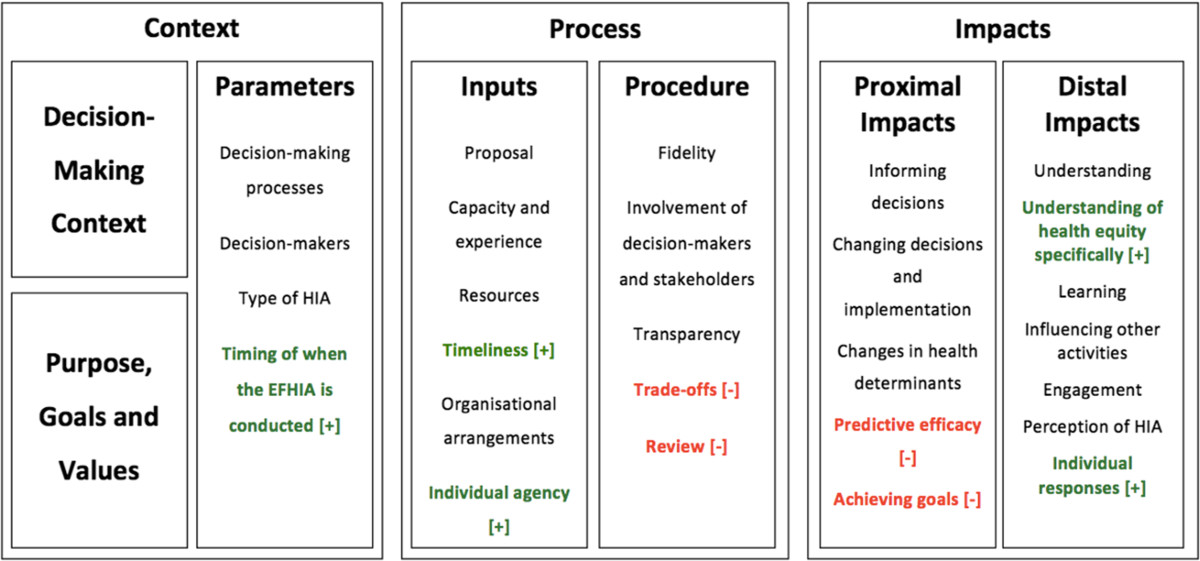
**Revised framework for evaluating the impact and effectiveness of equity focused health impact assessment.** Notes: Bold text indicates changed conceptual framework elements. Items with [+] and green text are new framework elements. Items with [−] and red text are existing framework elements that were not confirmed through this study.

The results illustrate that most of the items in the conceptual framework for evaluating the impact and effectiveness of HIAs (see Figure 1) were confirmed in relation to EFHIAs of health service plans, however some new items were identified through analysis and some existing items in the framework were not confirmed. These are outlined in Figure 2.

The first new item added as a parameter is the timing of when the EFHIA is conducted. Previously this had been described as time, which was a process factor. The EFHIAs in this study however showed that there was another distinct factor at play, which involved the point in planning and implementation at which the EFHIA was undertaken. The timing was often dictated by external decision-making factors and needs to be understood as a broader parameter under which EFHIAs are undertaken. Similarly timeliness has replaced time as an input into the EFHIA process in the revised conceptual framework because it is not just the time required to undertake the EFHIA but the timeframes of the broader planning and decision-making processes the EFHIA seeks to inform. The case studies showed that it was important to respond to these broader processes when scoping the EFHIAs.

Individual agency was added as an input into the process, because the extent to which many interviewees felt that they had a choice to be involved in the EFHIA or to commission it appeared to be closely related to the extent to which they were receptive to its recommendations or assisted the EFHIA process. This manifestation of agency appeared to take place at an individual level, particularly when the EFHIA was regarded as someone else’s idea or that someone else imposed their participation on them. This may also be linked to the increased focus on values and resource distribution that is specific to EFHIA, which may lead to examination of the values and assumptions underpinning planning processes.

Understanding of health equity was added under the distal domain as a new item because it was highlighted consistently throughout the interviews and documents analysed. This may be expected given EFHIA’s explicit equity focus. It relates to improved understandings of how plans may redress or exacerbate health inequities as well as specific equity issues that may arise in relation to potentially affected populations, for example the Good for Kids, Good for Life EFHIA showed how the original plan may have had a number of undesirable differential impacts on Aboriginal communities.

The other new distal impact that was added to the framework based on the analysis was Individual responses. These individual responses are both impacts themselves, i.e. the EFHIAs changed people’s individual responses and attitudes in several cases, but they also served to impede or facilitate other related impacts, i.e. individual responses led to the recommendations being discounted or rejected. In this way individual responses are both a distal impact and a kind of effect modifier; they are changed by the EFHIA but also change the EFHIA itself. The interview data in particular showed that individual responses were important. Even though all the interviews were conducted a year after the EFHIAs were completed, there were sustained impacts on the individuals interviewed. This may highlight the importance of humans in the EFHIA process, which seems axiomatic but may be easy to overlook.

The original version of the framework emphasised organisational and structural factors relating to HIA but this study highlighted that the involvement and engagement *of individuals* is important in mediating the perceptions of effectiveness. This emphasises that EFHIA cannot be fully evaluated in only procedural or structural terms. Individuals play an important role in determining the impact and effectiveness of EFHIAs but also HIAs in general.A number of factors were identified in the original conceptual framework that were not found or confirmed in this study (see Figure 2). They include trade-offs and review under the procedure domain and predictive efficacy and achieving goals under the proximal impact domain. These factors may still be important, they were just not confirmed within the context of this study. For example predictive efficacy may not be important as these were all voluntary decision-support EFHIAs not done to satisfy regulatory requirements.

### Implications for EFHIAs of health service plans

This study aimed to investigate:

1. What are the impacts of EFHIAs conducted on health sector plans?
2. How does EFHIA improve the consideration of equity in health planning?
3. What changes to the conceptual framework [74] are required to evaluate at the impact and effectiveness of EFHIAs, if any?The impacts of EFHIAs conducted on health service plans are broadly similar to those of HIAs, with some suggestions from the case studies in this study that they may have more direct impacts on understandings of health equity issues relevant to planning and implementation. It also has the potential to influence individual responses, though this is unpredictable and appears to be dependent on other factors such as the degree of agency and choice amongst those involved in the EFHIA. This is reflected in the revised conceptual framework (Figure 2).

EFHIAs appear to improve the consideration of equity in health planning, though this study is too contextually specific to demonstrate this systematically. The mechanism for improving consideration of equity through EFHIA appears to be linked to (i) promoting a clearer articulation of values that inform both the EFHIA and the broader decision-making process, (ii) promoting a clearer articulation of the purpose of the EFHIA and the proposal being assessed, and (iii) negotiating the nature of the learning desired from an HIA technical, conceptual and/or social learning, see [53, 107, 113–115].

The conceptual framework requires some changes to adapt to the context of EFHIA. These are outlined in the previous section. The most significant change is to include items recognising the role and importance of individuals engaged in the process, alongside the existing structural and procedural factors.

This study suggests that EFHIA has the potential to improve health service planning by enhancing consideration of health equity, but this is dependent on a number of factors. If there is not agreement about the purpose of the EFHIA and some degree of expressed agency on the part of individuals involved, through direct involvement in the EFHIA process and some degree of choice to be involved, the extent of learning from the EFHIA and its impacts may be limited. As such EFHIA may lead to different learning about health equity issues when compared with normal planning practice, but it may also need to be regarded as a collaborative learning process rather than as simply a document or one-off activity.

### Strengths and limitations of this study

This study focuses on the specific use of equity focused HIAs on health service plans in Australia and as such its findings are somewhat contextually bound. As mentioned in the background section, these EFHIAs were also conducted during a period of reform within the health system, though ongoing processes of change and reform increasingly reflect the reality of health service planning in most countries. These EFHIAs were also rapid in nature and did not aim to comprehensively assess all potential health impacts. It is worth noting though that (i) these are real EFHIAs that were scoped to meet the needs and time pressures of real policy and program decision-making, and (ii) this limitation applies to all HIA case studies.

The findings will have relevance to HIA practice in other sectors and in other countries however, as well as to those with an interest in health service planning. The use of HIA in relation to health sector proposals clearly remains relevant based on these case studies, particularly when they look at the potential health equity impacts of proposals.

## Conclusions

The case studies showed that the EFHIAs all had some impact on decision-making and implementation, though most clearly in relation to understandings of equity and options for modifying service plans to ensure this was addressed. Timing, individual agency and individual responses to the EFHIA were identified as factors influencing the impact of the EFHIAs. The case studies also showed that the conceptual framework for evaluating the impact and effectiveness of HIAs [74] has relevance to EFHIAs but requires some adjustment to account for EFHIAs’ emphasis on health equity and conceptual learning.

This study suggests EFHIA has the ability to enhance health service planning but this is dependent on a number of factors. In particular, if an EFHIA is to result in significant learning beyond technical learning [55, 107, 115] there may need to be shared understanding and agreement about the purpose of the EFHIA at an early stage in the process. For an EFHIA to lead to meaningful learning about health equity issues it may be necessary to regard it as a collaborative learning process integrated into planning activities rather than simply being a document or a discrete activity that occurs separate to planning. Studies comparing plans that have had EFHIAs conducted on them with similar plans that are the result of normal planning practice will be important in order to establish if this is the case.

## Electronic supplementary material

Additional file 1: CORE-Q Consolidated Criteria for Reporting Qualitative Research. Description of data: Table reporting on the characteristics of this qualitative research using standardised criteria. (PDF 67 KB)

Additional file 2: Description of this study using qualitative research review guidelines – RATS. Description of data: Table describing the context and attributes of this qualitative research using qualitative review guidelines (RATS). (PDF 96 KB)

Additional file 3: Coding nodes. Description of data: Table presenting the coding nodes developed through coding the qualitative data. (PDF 54 KB)

Below are the links to the authors’ original submitted files for images.

Authors’ original file for figure 1

Authors’ original file for figure 2

## Abbreviations

ABHI: Australian Better Health Initiative
COAG: Council of Australian Governments
EFHIA: Equity focused health impact assessment
EqIA: Equality impact assessment
HEIA: Health equity impact assessment
HIA: Health impact assessment
HNEAHS: Hunter New England Area Health Service
NSW: New South Wales
PHO: Public Health Officer
STI: Sexually transmitted infections.
