# Supplementary material for: Evaluating the impact of equity focused health impact assessment on health service planning: three case studies
Source: BMC Health Serv Res. 2014 Sep 5;14:371. doi: 10.1186/1472-6963-14-371 (PMC4161889; doi:10.1186/1472-6963-14-371)
Supplement: Supplementary file 1 — Additional file 1: CORE-Q Consolidated Criteria for Reporting Qualitative Research. Description of data: Table reporting on the characteristics of this qualitative research using standardised criteria. (PDF 67 KB) [file 12913_2014_3467_MOESM1_ESM.pdf]

Additional File 1: CORE-Q Consolidated Criteria for Reporting Qualitative Research

No	Item	Description
**Domain 1: Research Team and Reflexivity**		
	**Personal Characteristics**	
1	Interviewer / facilitator	Ben Harris-Roxas
2	Credentials	Bachelor of Social Work, Master of Policy and Applied Social Research, PhD (Public Health)
3	Occupation	Research and evaluation consultant, Conjoint Lecturer at the University of New South Wales
4	Gender	Male
5	Experience and Training	Has undertaken several qualitative studies, trained in interviewing, qualitative analysis and using NVivo
	**Relationship with participants**	
6	Relationship established	A relationship existed with 10 of the 14 interviewees prior to the interviews
7	Participant knowledge of the interviewer	Knew the researcher has worked on HIA and health equity for several years, the interviewer had contact with 10 of the 14 people interviewed through other activities than the EFHIA described
8	Interviewer characteristics	Is doing a PhD on EFHIA in health service planning
**Domain 2: Study Design**		

	**Theoretical Framework**	
9	Methodological orientation and theory	Interpretive description, case study methodology
	**Participant Selection**	
10	Sampling	Purposive
11	Method of approach	Emails (11 of 14) and phone calls (3 of 14)
12	Sample size	14
13	Non participation	No potential participants declined
	**Setting**	
14	Setting of data collection	Participants' workplaces, in person or on telephone
15	Presence of non-participants	No
16	Description of sample	A mix of those who developed the health service plan, those who conducted the EFHIA and those who were responsible for implementing its recommendations in each of the three case studies.
	**Data Collection**	
17	Interview guide	Provided in advance (see Table 2), piloted on 2 brief interviews not included in study
18	Repeat interviews	No
19	Audio/visual recording	Audio

20	Field notes	No
21	Duration	Mean 22 minutes, Range 16 minutes (min) to 40 minutes (max)
22	Data saturation	Yes, saturation across and within case studies was discussed by BHR and LK and by 14 interviews new categories and themes were not emerging.
23	Transcripts returned	No
**Domain 3: Analysis and Findings**		
	**Data Analysis**	
24	Number of data coders	Initial coding was done by BHR making two coding passes of all data. The first pass of all interviews coded against the existing conceptual framework, the second pass was free coded to identify different or emergent themes or items. The four longest interviews were also coded by FH, as someone with expertise in HIA, and Mark Harris, as someone with expertise in primary health care and service planning but not HIA, to ensure compatibility and soundness of coding and that there were no additional themes that had not already been identified.
25	Description of the coding tree	Yes, see Additional file 3
26	Derivation of themes	Themes were derived from an existing conceptual framework and also through open coding of interview transcripts and documents

27	Software	NVivo
28	Participant checking	No
	**Reporting**	
29	Quotations presented	Yes, selectively to illustrate analytic findings
30	Data and findings consistent	Yes
31	Clarity of major themes	Yes
32	Clarity of minor themes	No, the focus is on major analytic findings and higher-order changes to the conceptual framework
