# Supplementary material for: Evaluating the impact of equity focused health impact assessment on health service planning: three case studies
Source: BMC Health Serv Res. 2014 Sep 5;14:371. doi: 10.1186/1472-6963-14-371 (PMC4161889; doi:10.1186/1472-6963-14-371)
Supplement: Supplementary file 2 — Additional file 2: Description of this study using qualitative research review guidelines – RATS. Description of data: Table describing the context and attributes of this qualitative research using qualitative review guidelines (RATS). (PDF 96 KB) [file 12913_2014_3467_MOESM2_ESM.pdf]

Additional file 2: Description of this study against qualitative research review guidelines – RATS

Ask of the Manuscript	This paper
**R Relevance of study question**	
Is the research question interesting?<br>Is the research question relevant to clinical practice, public health, or policy?	Research question:<br>1. What are the impacts of EFHIAs conducted on health sector plans?<br>2. How does EFHIA improve the consideration of equity in health planning?<br>3. What changes to the conceptual framework are required to evaluate at the impact and effectiveness of EFHIAs, if any.<br>These questions explicitly address research priorities that have been identified in the literature.
**A Appropriateness of qualitative method**	
Is qualitative methodology the best approach for the study aims?	This study is a multiple retrospective case study of three completed EFHIAs. Data was collected through on semi-structured interviews with 14 participants and analysis of 7 reports and process documents (draft plans, EFHIA reports and revised plans) related to the EFHIAs and their implementation. Interviews were appropriate because this study seeks to understand perceptions of what changed through doing the EFHIAs and how this change occurred.
**T Transparency of procedures**<br>***Sampling***	
Are the participants selected the most appropriate to provide access to the type of knowledge sought by the	Purposive sampling of both case studies and of participants within case studies. The cases were selected to ensure they were EFHIAs of health service plans that had been conducted around the similar time (commencing in 2008), with similar levels of technical support and experience in EFHIA, and with differing perceptions of effectiveness, ranging from limited impacts on decision-making and

Ask of the Manuscript	This paper
study?<br>Is the sampling strategy appropriate?	implementation through to perceptions of significant impacts. Efforts were made to ensure that each case study included interviewees who were responsible for conducting the EFHIA, participated in the steering committee meetings, and who were responsible for acting on the EFHIA's recommendations.
Recruitment	
Was recruitment conducted using appropriate methods?	Participants were identified through documentation and initial discussions with people involved with each of the case studies.
**Is the sampling strategy appropriate?**	
Could there be selection bias?	All potential participants who were approached agreed to participate in the study. Participants were all actively involved in the EFHIA or in implementing its recommendations. No people were interviewed with limited involvement in the EFHIA case studies.
Data collection	
Was collection of data systematic and comprehensive?	The interviews were semi-structured. Though the content of each interview varied all interviewees were asked:<br>1) Tell us in your own words how the EFHIA was undertaken?<br>2) What changed as a result of doing the EFHIA?<br>3) Was the EFHIA a success? Why?<br>4) In general, what would make an EFHIA successful?

Ask of the Manuscript	This paper
	Additional related follow-up questions were asked based on the responses to these four questions.
Are characteristics of the study group and setting clear?	The 14 participants were all people who had all been involved in the EFHIAs or been responsible for considering and/or implementing the EFHIA's recommendations. 16 had backgrounds in health services, public health or health planning; 1 was a representative of an involved NGO.
Why and when was data collection stopped, and is this reasonable?	Data saturation was discussed between BHR and LK during the study. After 14 interviewees new themes both within an between case studies were not emerging.
Role of researchers	
Is the researcher(s) appropriate? How might they bias (good and bad) the conduct of the study and results?	BHR had existing relationships with 10 of the interviewees and conducted the interviews. The authors have all previously been involved in HIAs, which may influence their understanding and expectations about what will come out of conducting HIAs. To address this BHR's qualitative coding was checked by FH and Mark Harris, a researcher with a background in health services research and primary health care but not a history of involvement in HIA.<br>This study's methodology depended on existing relationships and the credibility of the researchers. Researchers without this background would have encountered greater reluctance from participants and organisations to take part in the study.
Ethics	
Was informed consent sought and granted?	Written consent was obtained from participants. A written information sheet about the study was provided to each participant along with details of ethics approval.
Were participants' anonymity and confidentiality ensured?	Participants' anonymity was ensured in the participant information form. Transcripts were de-identified and quotes have only been selectively used to minimise any possibility of participants being identified.

Ask of the Manuscript	This paper
Was approval from an appropriate ethics committee received?	Ethics approval was obtained from the University of New South Wales' Human Research Ethics Panel – Social/Health Research (9_08_121)
**S Soundness of interpretive approach** ***Analysis***	
Is the type of analysis appropriate for the type of study? Are the interpretations clearly presented and adequately supported by the evidence?	The analysis has two bases. Firstly it used an existing conceptual framework to identify and categorise potential factors and themes relating to the impact and effectiveness of the EFHIAs. Secondly it used an open coding approach to identify an additional or under-considered themes or factors. Where new interpretations are made these have been described in the body of the paper along with illustrative quotes from study participants' accounts.
Are quotes used and are these appropriate and effective?	Quotes have been used selectively to illustrate findings, partly to reduce repetition between quotes but also to minimise any risk of participants being identified through quotes.
Was trustworthiness/reliability of the data and interpretations checked?	Initial coding was done by BHR. The first pass of all 13 interviews and all 7 documents were coded against the existing conceptual framework in NVivo, the second pass was free coded to identify different or emergent themes or items. The four longest interviews were also coded by FH, as someone with expertise in HIA, and Mark Harris, as someone with expertise in primary health care and service planning but not HIA, to ensure compatibility and soundness of coding. This process found consistency in the themes that had been coded and resulted in no additional themes being identified.
Discussion and presentation	
Are findings sufficiently grounded in a theoretical or conceptual framework?	This study is presented with reference to the existing empirical literature and uses methods accepted for evaluating the effectiveness of HIAs. Further it is draws in an interpretive description theoretical

Ask of the Manuscript	This paper
Is adequate account taken of previous knowledge and how the findings add?	framework and seeks to test a conceptual framework for evaluating HIA.
Are the limitations thoughtfully considered?	The strengths and weaknesses of this study are discussed in the text.
Is the manuscript well written and accessible?	Yes, conforms with BMC Public Health manuscript requirements.
Are red flags present? These are common features of ill-conceived or poorly executed qualitative studies, are a cause for concern, and must be viewed critically. They might be fatal flaws, or they may result from lack of detail or clarity.	This study is based on interpretive description. As such it seeks to ground all interpretation in the accounts of participants and goes beyond description to provide an in-depth contextual description by drawing on the authors' interpretation and experience. It does this by synthesising, theorising and recontextualising rather than simply sorting and coding but this is required when conducting research on an evolving and practice-derived field such as HIA.
