# Supplementary material for: Evaluating the impact of equity focused health impact assessment on health service planning: three case studies
Source: BMC Health Serv Res. 2014 Sep 5;14:371. doi: 10.1186/1472-6963-14-371 (PMC4161889; doi:10.1186/1472-6963-14-371)
Supplement: Supplementary file 3 — Additional file 3: Coding nodes. Description of data: Table presenting the coding nodes developed through coding the qualitative data. (PDF 54 KB) [file 12913_2014_3467_MOESM3_ESM.pdf]

Additional file 3: Coding nodes

	Nodes	Sub Nodes
**Existing Conceptual Framework elements**		
	Broader Context	
	Distal Impacts	Engagement
		Influencing Other Activities
		Participatory Learning
		Perception of HIA
		Understanding
	Inputs	Capacity and Experience
		Organisational Arrangements
		Proposal
		Resources
		Time
	Parameters	Decision-Makers
		Decision-Making Processes
		Type of HIA
	Process	Involvement of Decision-Makers and Stakeholders
		Procedural Fidelity
		Review
		Trade-Offs
		Transparency
	Proximal Impacts	Achieving Goals
		Changes in Health Determinants
		Changing Decisions and Implementation
		Informing Decisions
		Predictive Efficacy
	Values, Purpose and Goals	

	Nodes	Sub Nodes
**Nodes that emerged from free coding**		
	Amenability to Change	
	Availability	
	Barriers	
	Changes during the HIA	
	Chaos	
	Cultural appropriateness	
	Demonstrating	
	Emotional Responses	Ambivalence
		Annoyance
		Comfortable
		Concerned
		Conflict
		Considered
		Criticism
		Disappointment
		Exclusion
		Frustrated
		Happy
		Resistance
		Support
		Suspicion
	Enablers	
	Equity Considerations in Planning	
	Evidence	
	Follow-Up	
	Improvements to HIA	
	Informed about what happened	
	Involvement of assessors	
	Memory	
	Nature of recommendations	
	Nature of report	
	Opportunities	
	Personalities	
	Planning vs HIA	
	Power	
	Prompt Debate	
	Rapid vs Comprehensive	

	Nodes	Sub Nodes
	Relationships	
	Subsequent changes	
	Successfulness	
	Taken Notice Of	
	Tangibility	
	Terms of Reference	
	Timing and Timeframes	
	Understanding at other points in the HIA process	
	Understanding of role	
	Volition	Someone else's idea
