# Supplementary figures and images for: Evaluating the impact of equity focused health impact assessment on health service planning: three case studies
Source: BMC Health Serv Res. 2014 Sep 5;14:371. doi: 10.1186/1472-6963-14-371 (PMC4161889; doi:10.1186/1472-6963-14-371)

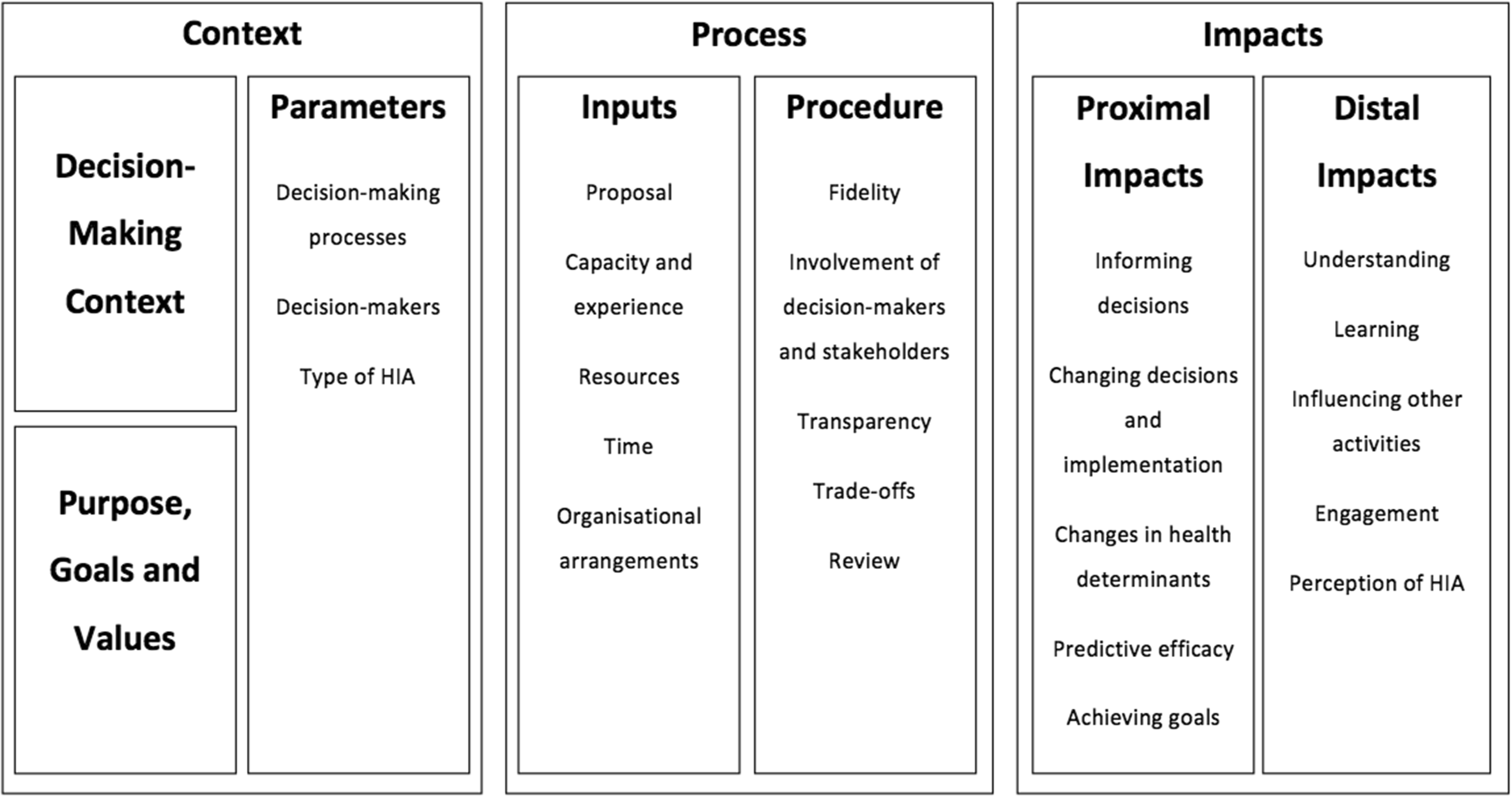

Supplement: Supplementary file 4 — Authors’ original file for figure 1 [file 12913_2014_3467_MOESM4_ESM.tif]

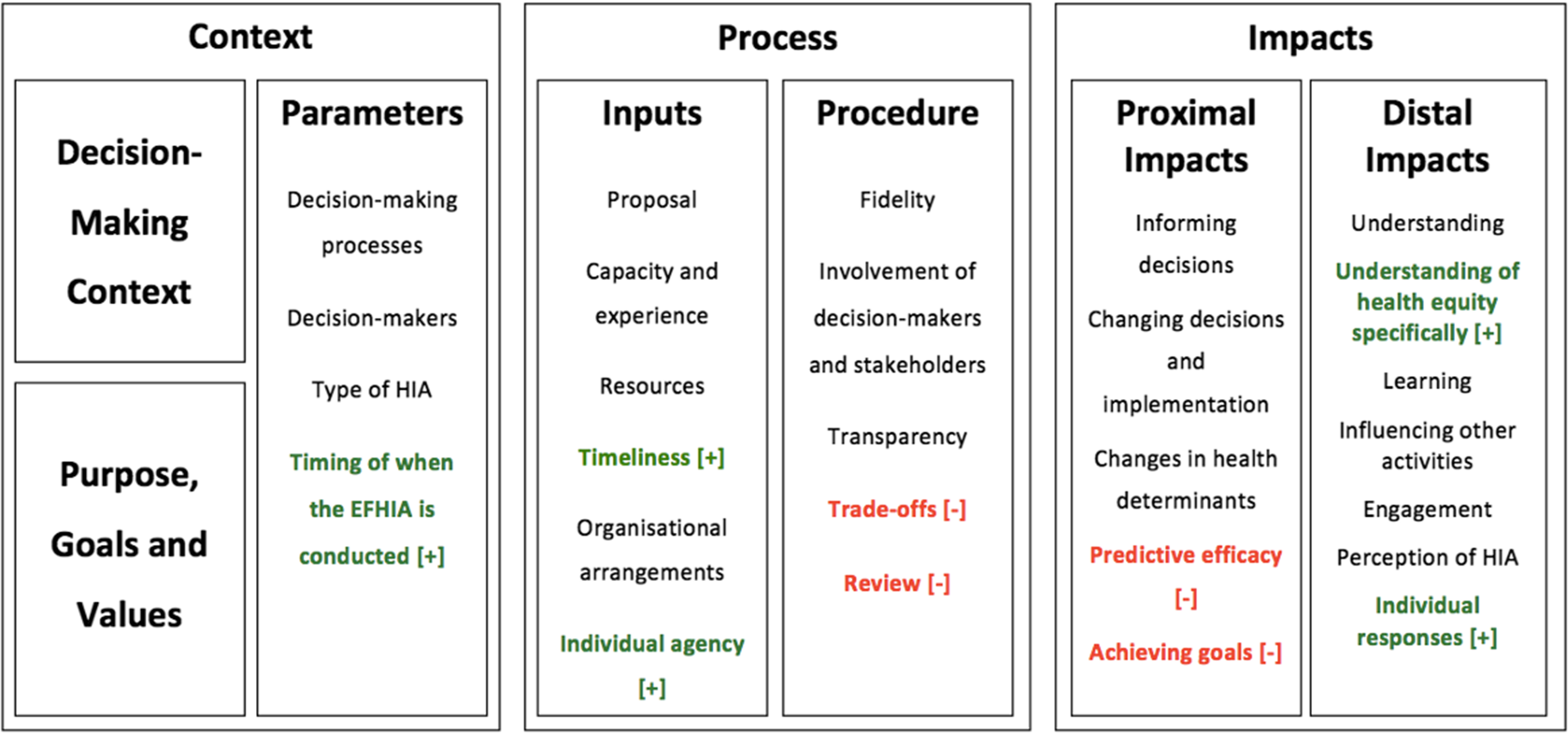

Supplement: Supplementary file 5 — Authors’ original file for figure 2 [file 12913_2014_3467_MOESM5_ESM.tif]
